# Preoperative Contrast-Enhanced MRI in Differentiating Glioblastoma From Low-Grade Gliomas in The Cancer Imaging Archive Database: A Proof-of-Concept Study

**DOI:** 10.3389/fonc.2021.761359

**Published:** 2022-01-17

**Authors:** Huangqi Zhang, Binhao Zhang, Wenting Pan, Xue Dong, Xin Li, Jinyao Chen, Dongnv Wang, Wenbin Ji

**Affiliations:** ^1^ Department of Radiology, Taizhou Hospital of Zhejiang Province affiliated to Wenzhou Medical University, Taizhou, China; ^2^ Department of Radiology, Taizhou Hospital, Zhejiang University, Taizhou, China

**Keywords:** gliomas, radiomics, MRI, histological grade, machine learning

## Abstract

**Purpose:**

This study aimed to develop a repeatable MRI-based machine learning model to differentiate between low-grade gliomas (LGGs) and glioblastoma (GBM) and provide more clinical information to improve treatment decision-making.

**Methods:**

Preoperative MRIs of gliomas from The Cancer Imaging Archive (TCIA)–GBM/LGG database were selected. The tumor on contrast-enhanced MRI was segmented. Quantitative image features were extracted from the segmentations. A random forest classification algorithm was used to establish a model in the training set. In the test phase, a random forest model was tested using an external test set. Three radiologists reviewed the images for the external test set. The area under the receiver operating characteristic curve (AUC) was calculated. The AUCs of the radiomics model and radiologists were compared.

**Results:**

The random forest model was fitted using a training set consisting of 142 patients [mean age, 52 years ± 16 (standard deviation); 78 men] comprising 88 cases of GBM. The external test set included 25 patients (14 with GBM). Random forest analysis yielded an AUC of 1.00 [95% confidence interval (CI): 0.86–1.00]. The AUCs for the three readers were 0.92 (95% CI 0.74–0.99), 0.70 (95% CI 0.49–0.87), and 0.59 (95% CI 0.38–0.78). Statistical differences were only found between AUC and Reader 1 (1.00 vs. 0.92, respectively; p = 0.16).

**Conclusion:**

An MRI radiomics-based random forest model was proven useful in differentiating GBM from LGG and showed better diagnostic performance than that of two inexperienced radiologists.

## Introduction

The tumor microenvironment could offer information that assists clinical decision-making.

According to the histological grade introduced by the World Health Organization (1), malignant gliomas are divided into low-grade gliomas (LGGs, grades 2–3) and glioblastomas (GBMs, grade 4) based on the tumor microenvironment. Survival varies significantly by grade across all glioma subtypes. GBMs have the poorest overall survival, with only 0.05%–4.7% of patients surviving 5 years post-diagnosis (2). Maximal surgical resection plays a central role in the management of gliomas. GBM tends to respond to postsurgical radiotherapy and chemotherapy due to its invasive nature, whereas postsurgical radiotherapy is associated with low benefit and risk of side effects in LGG (3–5). Pathological examination with invasive method is the gold-standard method used to differentiate between LGG and GBM (6).

Magnetic resonance imaging (MRI) has been widely used clinically to diagnose craniocerebral tumors because of its excellent soft-tissue resolution. However, distinguishing LGG from GBM by MRI scanning has low specificity. Yu et al. (7) found that the accuracy of the diagnostic performance correlated with the working experience of the radiologists. Imaging features can offer information regarding tumor homogeneity to distinguish LGG from GBM (8, 9).

Radiomics, extracted from computed tomography (CT) and MRI, could produce accurate robust evidence to assist clinical decision-making (10). Radiomics use high-throughput methods to extract and analyze qualitative information that cannot be assessed by visual inspection of clinical images on CT and MRI, as well as other images based on intensity, shape, size, and texture. Radiomics maximizes the information gained from clinical images and has been used in the diagnosis, treatment, and prognosis assessment of head and neck tumors (10–13). Recent studies have shown that MRI radiomics-based machine learning models perform well in predicting the histological grade and genetic mutations in glioma (14–16). However, an increasing number of published prediction models lack reproducibility evaluation (17).

The purpose of this study was to build a repeatable machine learning model based on contrast-enhanced MRI to predict the histological grade of glioma and provide more clinical information to improve treatment decision-making.

## Materials and Methods

### Data of Patients

MRI image acquisition and data set sampling: Our data were obtained from The Cancer Imaging Archive (TCIA) (https://www.cancerimagingarchive.net/). The inclusion criteria were as follows: 1) GBM and LGG collections that were identified, selected, and labeled by expert board-certified neuroradiologists (18, 19); 2) the preoperative baseline scans of these collections with MRI modalities of at least T1-weighted, T2-weighted, contrast-enhanced T1-weighted, and fluid attenuation inversion recovery (FLAIR) imaging were available; 3) basic clinical information and postoperative tumor pathology were available.

This study included 102 patients with GBM and 65 patients with LGG from eight independent centers in the TCIA database (19). The clinical information including sex, age, and images of preoperative MRI and tumor grading based on postoperative tumor pathology were collected (18, 20, 21). The patients were divided into two groups: the training set and the external test set. To improve the reproducibility of the model among the different centers, we used an institution-based approach to select the training set and the external test set to stabilize the model (22). Patients from the Thomas Jefferson University, Philadelphia, PA, USA (The Cancer Genome Atlas-76 and -CS), were used as the external test set, and those from other institutions were used as the training set (Appendix 1). A multicenter collaboration was undertaken to offer comparable results and minimize the potential for systemic bias.

### Image Preprocessing

All MRI images were preprocessed and uploaded to the TCIA library, including T1 images co-register, resampling (1 × 1 × 1 mm^3^), skull removal, smoothing, and Neuroimaging Informatics Technology Initiative format conversion (18). The Oxford Centre for Functional MRI of the Brain (FMRIB) Linear Image Registration Tool (FLIRT) of the FMRIB Software Library (FSL) was used for co-registration. All preoperative MRI images were co-registered to the same T1 anatomic template using the affine registration method. The intensity of the non-uniformities of the images was not corrected, as the application of any non-parametric, non-uniform intensity normalization algorithm eliminated the T2-FLAIR signal.

### Image Segmentation

Image segmentation of MRI was completed with a computer-aided method named GLISTRboost (23) and subsequently corrected manually. The lesion was segmented into edema area, tumor contrast-enhanced area, and non-contrast-enhanced area. After segmentation, the area was revised and evaluated repeatedly by multiple experts until an agreement was reached (18). According to the TCIA description, using the gold-standard method, the segmentation results were widely recognized and ensured the feasibility if a cross-study comparison was done. Based on the segmentation label, we merged the tumor contrast-enhanced and non-contrast-enhanced areas as the region of interest (ROI) and then named it as the tumor area. The unit volume of the tumor component is 1 mm^3^/voxel. Since clinicians may not be able to distinguish the small tumor necrosis areas accurately, separate them from other components, and delineate them finely, we divided the entire tumor into tumor and edema areas after considering the application of the model.

### Feature Extraction

**Figure 1:**
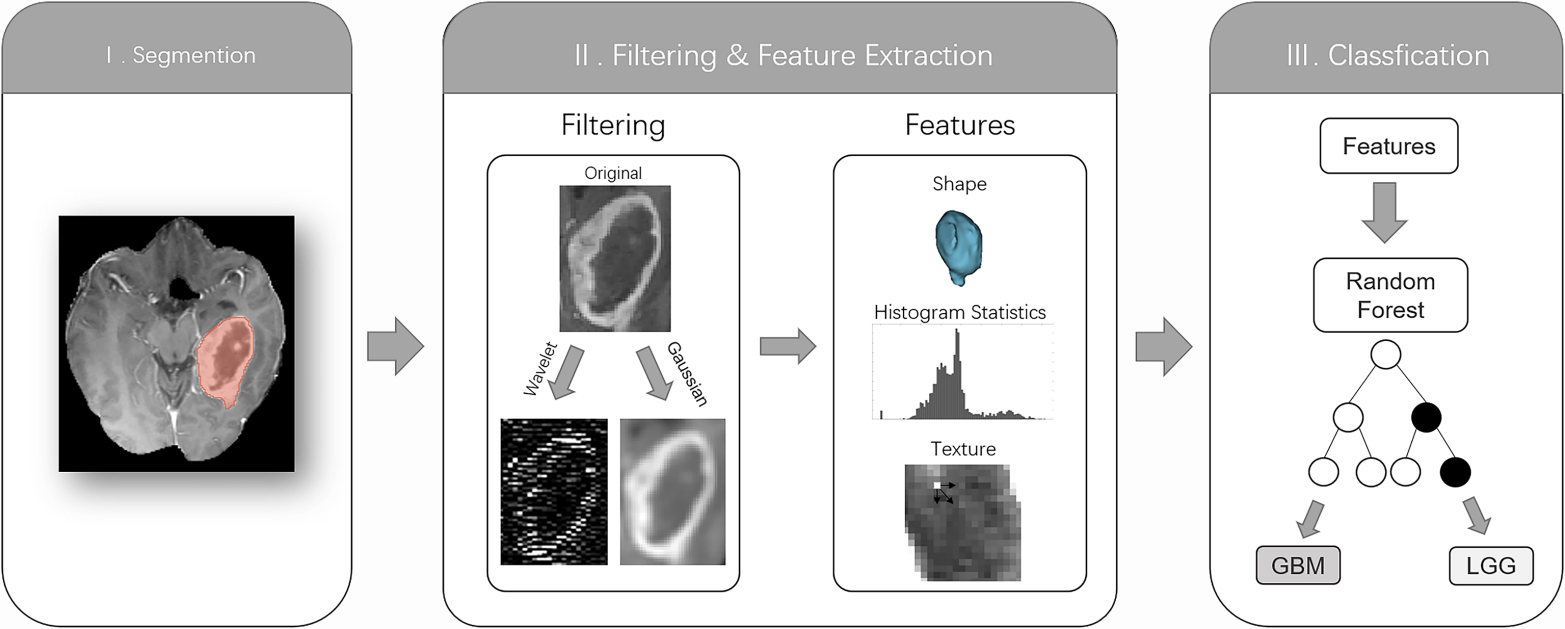
The radiomics workflow comprised three steps: segmentation of the tumor core (TC) on the contrast-enhanced MRI; image filtering and feature extraction feature histogram statistics, shape, or texture; and training a random forest classification algorithm based on features to distinguish low-grade gliomas from glioblastomas based on histopathological reference standards.

Radiomics features were extracted and filtered from segmented ROIs. The model was then verified using an external test set, and the radiomics process is shown in **Figure 1**. Due to its superior performance in the preliminary experiment (Appendix 2), CE-MRI was used to extract the radiomics features. Therefore, we used the PyRadiomics (V3.0.1; Harvard Medical School; https://github.com/Radiomics/pyradiomics) (24), an open-source Python package, and directly extracted quantitative features on the voxels of the tumor area segmented on the CE-MRI. In the process of setting the parameters on the PyRadiomics package, we normalized the graphics (normalized: true normalized Scale: 100) and then resampled the graphics to a voxel size of 2 × 2 × 2 mm to standardize the voxel space and set the bin width to 5 for discrete voxel intensity to reduce image noise and normalize image intensity (more specific parameter settings are shown in Appendix 3). The image is reconstructed by wavelet and log. The radiomics features are mainly divided into three categories, namely, first-order features, shape features, and Gray Level Co-occurrence Matrix (GLCM), Gray Level Run Length Matrix (GLRLM), Gray Level Size Zone Matrix (GLSZM), and Gray Level Dependence Matrix (GLDM) (24). These extracted radiological features were in line with the feature definition described by the Imaging Biomarker Standardization Initiative. We used 13 image filters for image transformation and extracted quantitative image features, including 18 first-order statistical features, 14 characteristic shapes (including size), and 68 texture features. A total of 1,132 [(18 + 68) 13 + 14] feature-filter combinations were built and named as features.

### Feature Selection

To differentiate between LGG and GBM, the Pearson correlation analysis was used to analyze feature reduction. Features could be retained with a correlation coefficient greater than 0.8. This feature selection method, based on paired feature correlation, was used to improve the machine learning training process and optimize the feature interpretability. As a result, 83/1,132 (7.3%) features were retained while building the radiomics model.

### Model Establishment

The entire code we used during the model establishment is publicly available on the development platform Github (https://github.com/pwesp/random-forest-polyp-classification) (25). After 142 lesions were divided into LGG or GBM based on histopathological examination, Random Forest classifier was used to construct a radiomics model. Random Forest Classifier class of the sklearn.ensemble library [scikit-learn Python machine learning library (26), version 0.22] had 1,000 trees (n _ estimators = 1,000) and other default parameters, 83 features in the training set made up of 142 gliomas. After machine learning, Random Forest Classifier class predicted each lesion as LGG or GBM. Bootstrap resamples of the entire training data were used to train each decision tree in the random forest. In the random forest trees, binary decision-making learns a randomly selected subset of features on a single node. This double randomness helps grow independent decision trees as much as possible; thus, “when the number of trees increases, the generalization error will almost certainly converge to a limit” (27). The implementation of the scikit-learn random forest follows the method used by Breiman et al. (27), with one exception: it combines classifiers by averaging the probability predictions of the classifiers instead of allowing each classifier to vote for a class. Compared with other machine learning algorithms, random forests are robust to outliers and noise (27, 28). The remaining training set samples used to train a tree are used to self-evaluate the corresponding trees and form the “out-of-bag” errors measuring the prediction error of random forests (27). In addition, the scikit-learn random forest provided an internal evaluation of the relative importance of the features, reflecting how much the degree prediction of the training model (GBM vs. LGG) relies on a specific feature relative to all others. We used it to estimate the relative importance of the features among the 83 features used in the establishment of the model and performed correlation tests on the top 15 features. Subsequently, the differences in age and the first and second most relative important features were compared in the training and external test sets.

### Comparison of Diagnostic Performance Between Model and Radiologists

**Figure 2:**
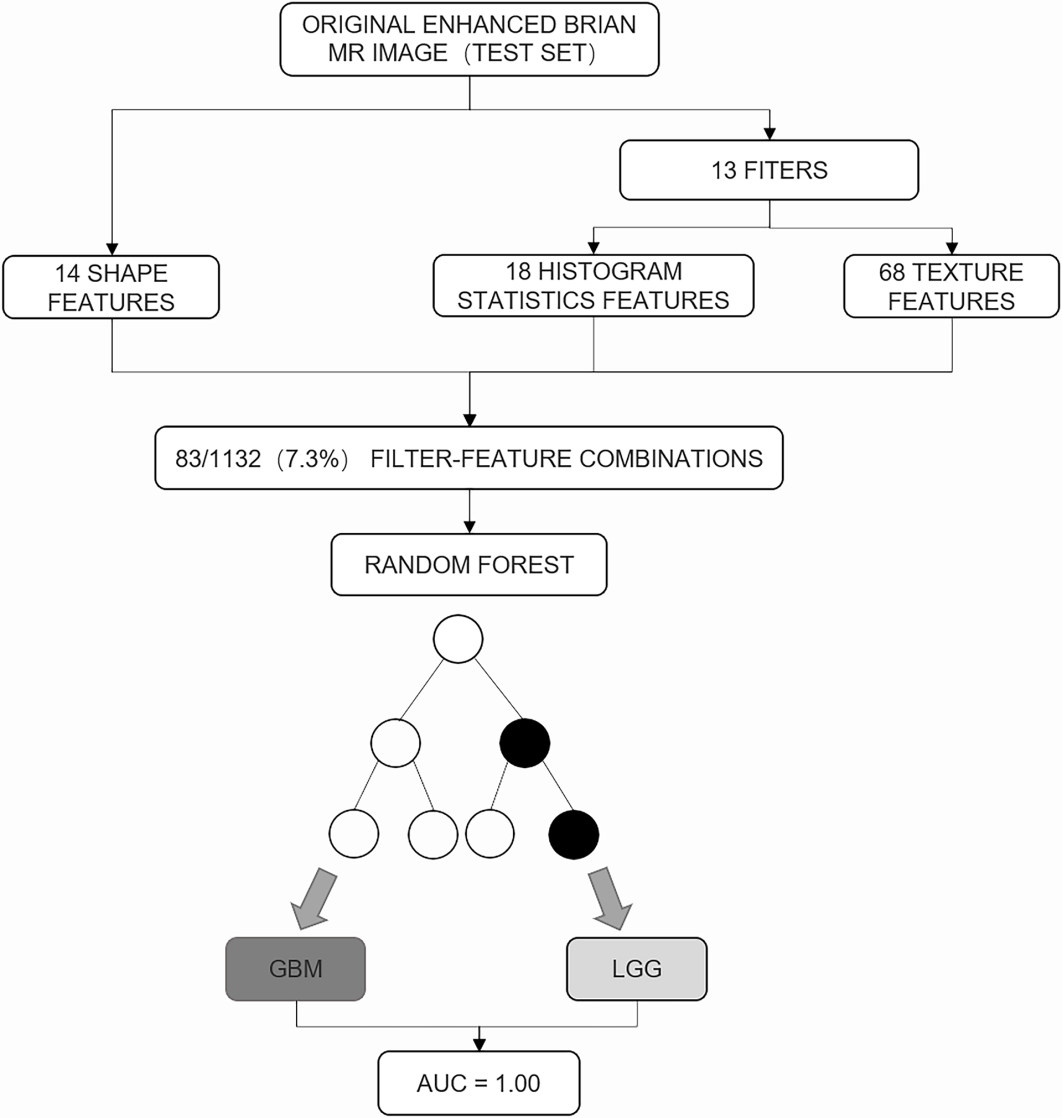
The random forest analysis of the test set. A total of 83 of 1,132 (7.3%) feature filter combinations were extracted from images of the test set using different image filters (n = 13) and image features characterizing shape (n = 14), histogram statistics (n = 18), or texture (n = 68). On the basis of these filter feature combinations, the trained random forest classifier was used to predict the low-grade glioma and glioblastoma. Prediction performance was quantified using area under the receiver operating characteristic curve (AUC).

The random forest analysis of the test set is shown in **Figure 2**. To compare the diagnostic performance of the prediction of LGG and GBM between the radiologist and the model, we also selected three radiologists (reader 1, neuroradiologist with 15 years of radiographic experience; reader 2, neuroradiologist with 1 year of radiographic experience; and reader 3, non-neuroradiologist with 3 years of radiographic experience) to independently evaluate 25 cases of glioma in the external test set on T1, T2, FLAIR, and T1 contrast-enhanced MRI. These radiologists assessed the size of the tumor, edge of the tumor, border of the tumor, peritumoral edema, degree of enhancement, necrosis, and other characteristics. All patients were diagnosed with grade 2, 3, and 4 gliomas according to the histological grade of the World Health Organization. The grade 2 and 3 gliomas were regarded as LGGs, and the grade 4 gliomas were regarded as GBMs. The three radiologists were blinded to the patients’ clinical data.

### Statistical Analysis

Statistics on the training and external test sets were independent. All statistical calculations were performed using Python (version 3.7.0) and MedCalc (version 19.0; MedCalc Software). The differences between the demographic data of the training set and those of the external test set were evaluated. The receiver operating characteristic curve was used to predict the diagnostic performance of the random forest model and radiologists. The cutoff value was preset using the maximum Youden index (29) from the training set, and the difference between ROCs was compared using the DeLong test (30); statistical significance was set at p < 0.05.

## Results

### Comparison of Patient Characteristics Between Training Set and Test Set

Patient characteristics: A total of 142 patients were included in the training set, of which 64 were male (54.9%, 52.3 years ± 16.0), 88 had GBM (61.9%), and 54 had LGG (28 were grade 3, and 25 were grade 2). According to the more detailed histological classification, oligodendroglioma (23 cases), astrocytoma (14 cases), oligoastrocytoma (17 cases), IDH1 mutant (33 cases), and IDH1 wild type (67 cases) were identified. The external test set included 25 patients, of which 13 were male (53.4 years ± 12.6), 14 had GBM (61.9%), and 11 had LGG (14 were grade 4, eight were grade 3, and three were grade 2). There were three cases of oligodendroglioma, seven cases of astrocytoma, 11 cases of oligoastrocytoma, four cases of IDH1 mutant type, and 16 cases of IDH1 wild type. Baseline data did not show a statistically significant difference between the training and external test sets (**Table 1**; p > 0.05).

**Table 1:** Clinical characteristics in the training and external test sets.

	Training Set (n = 142)	External Test Set (n = 25)	p-value
Age (years)*	52.3 ± 16.0	53.4 ± 12.6	0.72^a^
Gender (male/female)	78/64	13/12	0.79^b^
GBM/LGG	88/54	14/11	0.36^b^
G4/G3/G2	88/28/25	14/8/3	0.37^b^
Histology Glioblastoma/oligodendroglioma/astrocytoma/oligoastrocytoma	88/23/14/17	14/3/7/1	0.07^b^
IDH1 mutation/IDH1 wild type	48/68	7/18	0.10^b^

a Student’s t-test.

b Chi-square test.

TCGA, The Cancer Genome Atlas; IDH1, isocitrate dehydrogenase 1; SE, standard error.

*Age values are means ± standard deviation.

### Radiomics Feature Reduction

As mentioned above, we screened 83 features that were finally incorporated into the model from 1,132 extracted features, including six first-order statistical features, five characteristic shapes (including size), 13 texture features, and 59 high-order features after wavelet transform and log transform.

### The Diagnostic Performance of Radiomics Model

**Figure 3:**
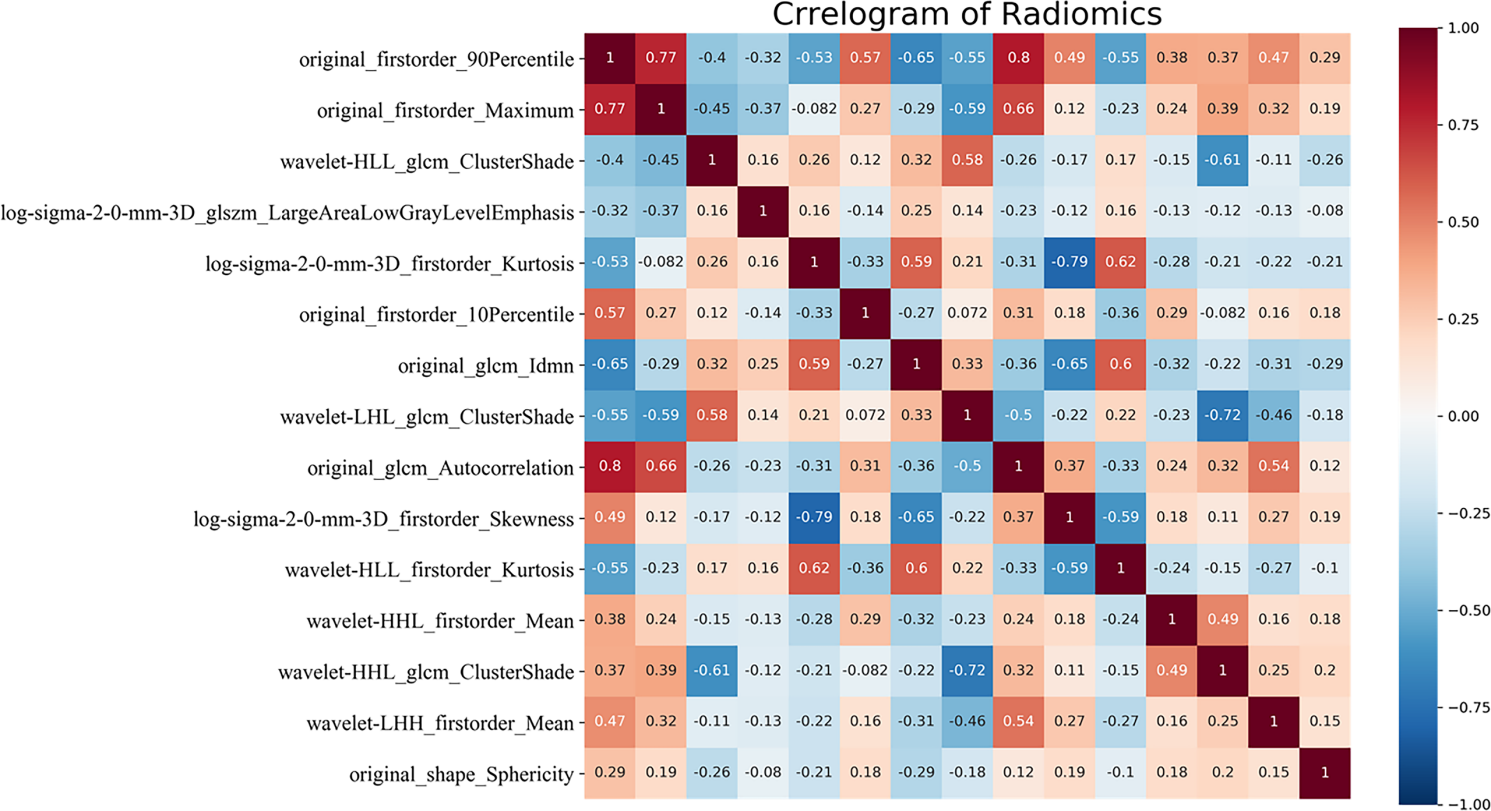
Correlation heatmap of the top 15 features of relative importance. glcm, gray level co-occurrence matrix; glszm, gray level size zone matrix; H, high-pass filter; L, low-pass filter; 3D, three-dimensional. HHH, LHH, LHL, LLH, and LLL indicate wavelet transform bands in the X, Y, and Z axes, respectively.

After constructing the random forest model constructed, the AUC of the radiomics model to distinguish between LGG and GBM in the training set was 0.930. The maximum Youden index (0.610) was selected as the cutoff value, and the sensitivity and specificity of the model obtained were 0.880 and 0.910, respectively. The Youden index is sensitivity + specificity -1, when its range is 0–1.0, indicating that the model’s ability is perfect. In the external test set, when the AUC was 1.000 and the cutoff value was 0.610, the sensitivity and specificity were 1.000 (14/14) and 1.000 (11/11), respectively. The random forest model calculated the relative importance of the 83 features (Appendix 1). The top 2 features were original_firstorder_90Percentile: 0.129, original_firstorder_ Maximum: 0.051. The heatmap of correlation among 15 features has been shown in **Figure 3**. The correlation coefficient was 0.77 between original_firstorder_90Percentile and original_firstorder_Maximum.

### Comparison of Diagnostic Performance Between Random Forest Model and Radiologist

In the external test set, the AUCs were 1.0 (95% CI 0.86–1.00), 0.92 (95% CI 0.74–0.99), 0.70 (95% CI 0.47–0.87), and 0.59 (95% CI 0.38–0.59) for the radiomics model, readers 1, 2, and 3, respectively. No difference was noted in the AUC between the imaging radiomics model and the senior physicians (p = 0.16). However, statistical significance was found between the model and younger physicians (p < 0.001 and p = 0.001, respectively) (**Table 2**).

**Table 2:** Diagnostic performance of the radiomics model and the three readers in the external test set.

Parameter	Radiomics Model	Reader 1	Reader 2	Reader 3
AUC	1.00 [0.86, 1.00]	0.92 [0.74, 0.99]	0.70 [0.49, 0.87]	0.59 [0.38, 0.78]
Sensitivity(%)	100 (14/14) [0.77, 1.00]	93 (13/14) [0.66, 1.00]	86 (12/14) [0.57, 0.98]	64 (9/14) [0.35, 0.87]
Specificity(%)	100 (11/11) [0.77, 1.00]	91 (10/11) [0.59, 1.00]	55 (6/11) [0.23, 0.83]	55 (6/11) [0.23, 0.83]

Data in parentheses are numbers of patients, with 95% CIs in brackets. There were no differences between the AUCs of the radiomics model and an experienced radiologist (p = 0.16). However, the radiomics model outperformed those of readers 2 and 3 (p < 0.001 and p = 0.001, respectively).

### Difference of Diagnostic Performance Between Clinical Parameter and Radiomics Features

**Figure 4:**
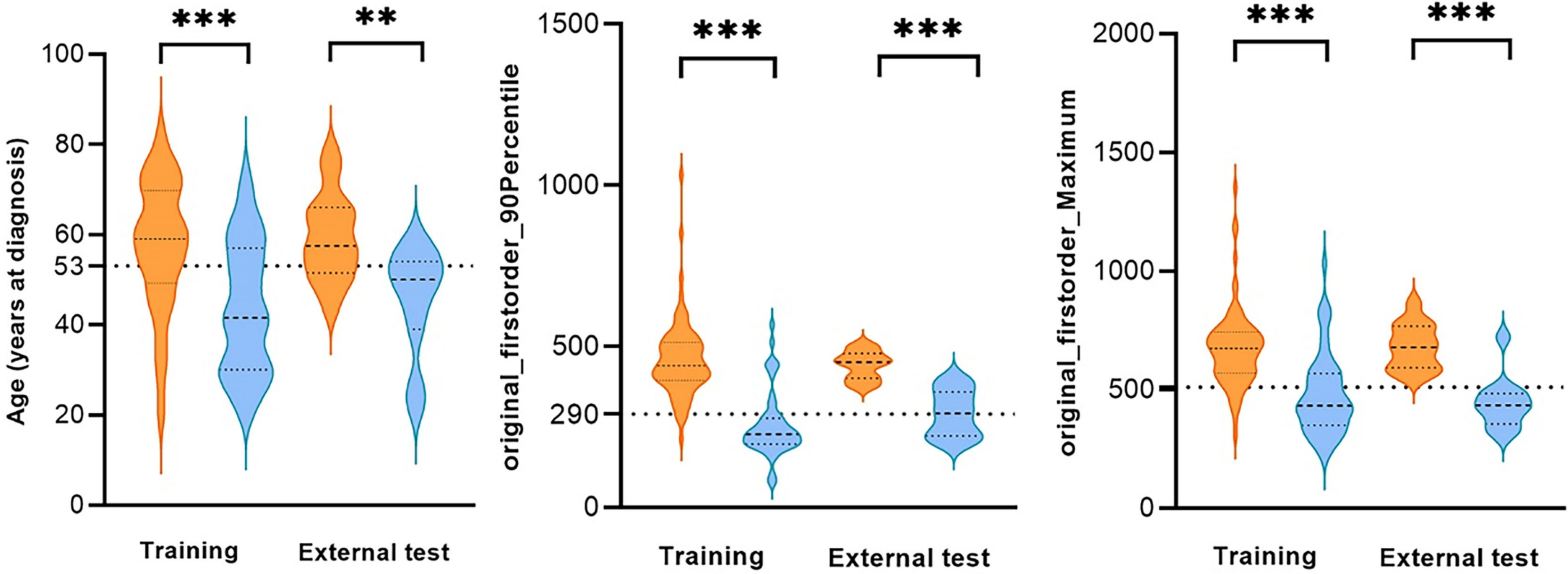
Comparison of age and the first and second relative important features of glioblastoma (GBM) and low-grade glioma (LGG) in the training set and external test set. **p < 0.01, ***p < 0.001, dotted line: cutoff value.

**Figure 5:**
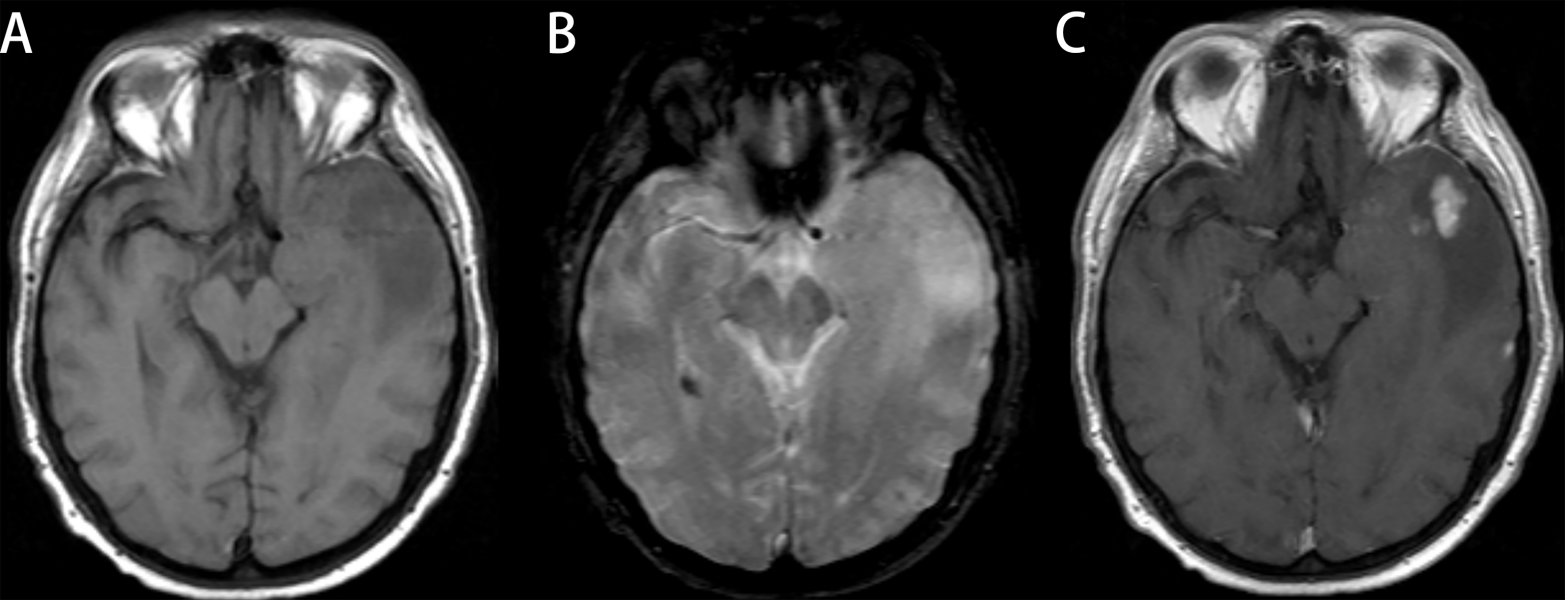
Glioblastoma (GBM) in a 78-year-old man with a license named TCGA-76-6193 (TCGA-76-6193). **(A)** Axial T1-weighted MR images demonstrate an area of high signal intensity in the left temporal region with adjacent edema. **(B)** Axial T2-weighted MR image reveals slightly high signal in the lesion. **(C)** Axial T1-weighted MR image with contrast material showed significant and heterogeneous enhancement in the lesion. All three readers diagnosed the lesion as low-grade glioma (LGG). Radiomics predict_proba_GBM was 0.701 (cutoff value = 0.610), and the radiomics model diagnosed it as GBM.

**Figure 6:**
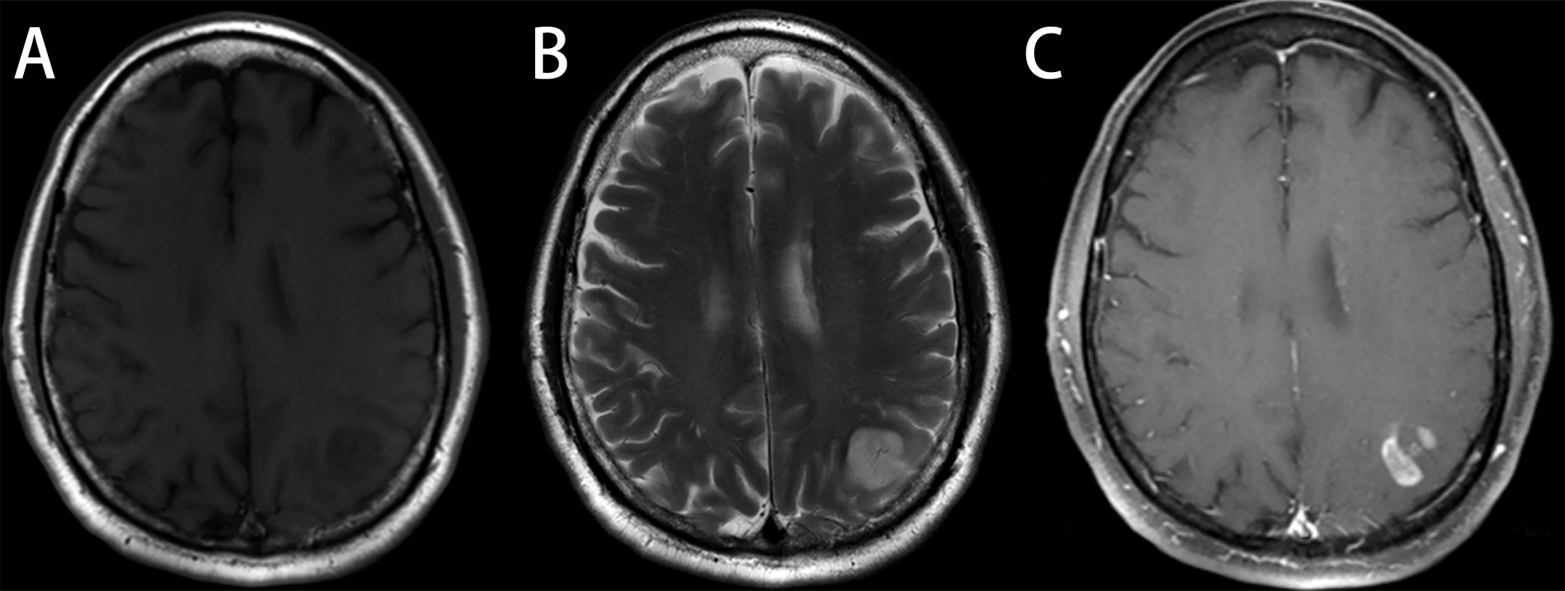
Astrocytoma, grade 3, in a 48-year-old man with a license named TCGA-CS-6188 (TCGA-CS-6188). **(A)** Axial T1-weighted MR images demonstrate an area of heterogeneous low signal intensity in the left occipital region with adjacent edema. **(B)** Axial T2-weighted MR image reveals a heterogeneous high signal in the lesion. **(C)** Axial T1-weighted MR image with contrast material showed significant “flower lace” enhancement in the lesion. Reader 1 diagnosed the lesion as glioma, grade 3, and the diagnosis of reader 2 and reader 3 was glioblastoma (GBM). Radiomics predict_proba_GBM was 0.512 (cutoff value = 0.610), and the radiomics model diagnosed it as low-grade glioma (LGG).

In the training set, 88 patients had GBM (57.6 years ± 1.5) and 54 had LGG (43.7 years ± 2.0). The difference between GBM and LGG in terms of age and the first and second relative important features are shown in **Figure 4**. The cutoff values of age, “original_firstorder_90Percentile,” and “original_firstorder_Maximum” were >53 years, >290, and >510, respectively. These three variables yielded overall AUCs of 0.812 [95% confidence interval (CI): 0.606–0.939], 0.968 (95% CI: 0.808–1.000), and 0.942 (95% CI: 0.770–0.996), respectively. The respective AUCs for the other 81 radiomics features are shown in Appendix 1. In the external test set, 14 patients had GBM and 11 had LGG. The cutoff values were set the same as the training set. The AUCs for age, “original_firstorder_90Percentile,” and “original_firstorder_Maximum” were 0.721 (95% CI: 0.507–0.880), 0.727 (95% CI: 0.514–0884), and 0.955 (95% CI: 0.788–0.998), respectively. **Figures 5**, **6** show two examples of different opinions regarding LGG/GBM between the radiologists and radiomics model.

## Discussion

We used the data of patients with LGG or GBM from the TCIA database to construct a repeatable random forest model based on preoperative contrast-enhanced MRI. The AUCs in the training set and the external test set for identifying the LGG and GBM were 0.93 and 1.00, respectively, and no difference was observed in the AUC between the imaging radiomics model and senior doctors (p = 0.16).

Judging the degree of malignancy, which is important for clinicians, is challenging when a glioma is suspected. The random forest model obtained in this study was used to identify GBM and LGG. The AUC in the training set was 0.93, and it reached 1.0 in the external test set. A previous study (7) used qualitative imaging features to distinguish the histological grades of gliomas. The AUCs of edema and non-contrast enhancement were 0.803 and 0.753, respectively. Qualitative assessment depends on the experience of the radiologists, and comparability cannot be guaranteed. Numerous studies have been conducted on the identification of high-grade gliomas and LGGs based on radiomics. Cao et al. (8) found that the brain regions where gliomas occur and tumor components can distinguish benign and malignant gliomas. The AUC of the model in the training set was 0.997 and that in the external test set was 0.90. Their research only focused on the morphological features. The model exhibited good performance; however, the necessary process of image co-registration required manual calibration to ensure accuracy, which may decrease the feasibility of clinical application. Lambin et al. (10) emphasize that the principal challenges of applying radiomics to clinical practice are the optimal collection and integration of diverse multimodal data (for example, the multiparametric MRI data) and reproducibility of the models. Takahashi et al. (31) used a machine learning model based on diffusion kurtosis and tension imaging to identify GBM and LGG. In the external test set, the AUC reached 0.98 in the comprehensive model, but the sample size was too small, with only 55 cases. In comparison, we only used the sequence of T1 contrast-enhanced MRI, which may have better general applicability, as it may be difficult to acquire high-quality diffusion tensor imaging images in grassroots hospitals.

In the radiomics process, we set the normalization scale = 100 and the bin width = 5 to make the bins equal to approximately 100. Bins between 16 and 128 were ideal in the subsequent analysis, which also made the images obtained comparable between different scanning machines. In addition, skull-strip may potentially eliminate interference with the analysis of the ROI’s radiomic features.

In the model obtained in this study, the “original_firstorder_90Percentile” feature contributed considerably to the identification ability of the model, and its relative importance reached 0.129. The feature “original_firstorder_90Percentile” reflected 90% of the voxel intensity of the image after skull-strip, resampling, and normalization. The feature “original_firstorder_Maximum” (the maximum value of the image voxel intensity) after the similar process described above, which is relatively important, belonged to the feature describing the voxel intensity of the image as the feature “original_firstorder_90Percentile.” These two features provided an almost 1/5 relative importance. Since malignant gliomas may have high expression of angiogenic factors, such as vascular endothelial growth factor and angiotensin, the tumors often have rich vascular components. In a previous study (32), Ang-II was reported to be highly expressed in malignant glioma cells, the necrotic part (with degeneration of the blood vessels), and the tissues surrounding the tumor (indicating angiogenesis) and was related to the formation of immature vessels in the tumor. Since a malignant glioma destroys the blood–brain barrier and has rich vascular components, it is not difficult to explain why the tumor can take up an abundant amount of contrast agent in contrast-enhanced MRI and show a relatively high level of enhancement. Several important radiomics features obtained in this study also validated the findings of Yu et al. (7). The proportion of non-enhancing tumors is an independent predictor of GBM (grade 4). Liu et al. (33) analyzed perfusion imaging of GBM and found a subgroup of tumors that could benefit from antiangiogenic therapy. This group of patients had more abundant angiogenesis pathways and a worse prognosis. In general, the degree of tumor enhancement is of great significance to distinguish between high-grade gliomas and LGGs. Although imaging radiomic characteristics are used, the degree of tumor enhancement judged by visual inspection may also be a point that requires particular attention. A higher degree of enhancement in the solidity of the tumor often suggests that the glioma belongs to a higher histological grade category. Compared with other studies, the proposed model is generalizable. Several radiomics models with good performance or interpretable feature labels are available; however, their applicability and repeatability are difficult to guarantee. The radiomics quality score proposed by Lambin et al. (10) also discussed the above. Technology application is inseparable from standardization, and the absence of standards means that quality is not guaranteed in the promotion of technology. The data in this study were obtained from public databases, which guaranteed the verifiability and relative simplicity of the results. The signal strength of MRI is largely affected by the magnetic field strength. The extraction of radiomic features was based on a series of image preprocessing to ensure that the images with various scan parameters from different centers were comparable. It is difficult to accurately distinguish and segment tumor enhancement components, necrotic components, and edema areas. We combined necrosis and enhancement area and then combined them into the tumor area, which may make it more feasible for clinical application in the future.

Our study has several limitations. First, all our data were obtained from public databases. The ROI of the images was drawn semiautomatically and corrected by experienced experts. The gold-standard segmentation label also brought corresponding challenges while ensuring segmentation accuracy, and realizing the gold-standard segmentation is not easy, but the development of technology of automatic segmentation for brain image may give a feasible settlement (34, 35). The impact of individual differences in the image segmentation process was not evaluated in our research. No difference was found in the results of this study because the segmentation was regarded as the gold standard. Nevertheless, considering further application of the model, the impact of individual differences cannot be ignored. Therefore, using other data is necessary to perform external verification of the radiomics model, which is currently in progress. Second, we divided the training set and the external test set based on the organization, and only one center met the requirements of GBM : LGG = 1:1. The non-random selection of the external test set may introduce a potential selection bias in the research results. The number of samples in the external test set was only 25. Whether the small external test set can play a role in discovering model overfitting remains unclear. The latest World Health Organization classification of gliomas incorporated the mutation status of IDH-1 based on histology (36). We did not consider the mutation status of IDH-1 in our study because we considered that molecular information only played a supplementary role. When grading the degree of malignancy, the difference in histology is still important. The study by Hartmann et al. (37) analyzed the impact of IDH-1 mutation status and histological grade on the prognosis of patients with glioma. The results showed that regardless of the IDH-1 mutation status, higher histological grades often indicate worse prognosis.

## Conclusions

Our model is generalizable. The performance of the model was comparable to that of experienced radiologists, and it was better than that of inexperienced radiologists. Our model can reduce the workload of radiologists and improve the diagnostic accuracy for glioma.

## Data Availability Statement

The original contributions presented in the study are included in the article/supplementary material. Further inquiries can be directed to the corresponding author.

## Ethics Statement

Written informed consent was obtained from the individuals for the publication of any potentially identifiable images or data included in this article.

## Author Contributions

HZ, BZ, WP, and XD: conception and design. HZ, XL, JC, DW, and WJ: collection and assembly of data. HZ: data analysis and interpretation. BZ and WP: article writing. All authors contributed to the article and approved the submitted version.

## Funding

This research did not receive any specific grant from funding agencies in the public, commercial, or not-for-profit sectors.

## Conflict of Interest

The reviewer ZD declared a shared affiliation with one of the authors XD to the handling editor at the time of review.

The remaining authors declare that the research was conducted in the absence of any commercial or financial relationships that could be construed as a potential conflict of interest.

## Publisher’s Note

All claims expressed in this article are solely those of the authors and do not necessarily represent those of their affiliated organizations, or those of the publisher, the editors and the reviewers. Any product that may be evaluated in this article, or claim that may be made by its manufacturer, is not guaranteed or endorsed by the publisher.

## Abbreviations

MRI, magnetic resonance imaging; GBM, glioblastoma; LGG, low-grade glioma; AUC, area under the receiver operating characteristic curve; CI, confidence interval; TCIA, The Cancer Imaging Archive; ROI, region of interest.
